# ﻿Taxonomic review of Manocoreini with description of a new species from China (Hemiptera, Heteroptera, Coreidae)

**DOI:** 10.3897/zookeys.1152.98234

**Published:** 2023-03-09

**Authors:** Yanyan Zhou, Huaxi Liu, Wenjun Bu, Zhiqiang Li

**Affiliations:** 1 Guangdong Key Laboratory of Animal Conservation and Resource Utilization, Guangdong Public Laboratory of Wild Animal Conservation and Utilization, Institute of Zoology, Guangdong Academy of Sciences, No.105 Xingangxi Road, Guangzhou, 510260, Guangdong, China Institute of Zoology, Guangdong Academy of Sciences Guangzhou China; 2 Department of Life Sciences, Silwood Park Campus, Imperial College London, SL5 7PY, Ascot, UK Imperial College London Ascot United Kingdom; 3 Department of Life Sciences, Natural History Museum, SW7 5BD, London, UK Natural History Museum London United Kingdom; 4 Institute of Entomology, College of Life Sciences, Nankai University, Weijin Road 94, 300071, Tianjin, China Nankai University Tianjin China

**Keywords:** Coreoidea, *Manocoreus*, Oriental Region, taxonomy

## Abstract

In the present paper, all seven species of Manocoreini are reviewed, and a new species *Manocoreushsiaoi***sp. nov.** is described from Guangxi, China. Photographs of habitus of all species, and detailed structures of the new species and type species of *Manocoreus* Hsiao, 1964 are provided. All species of Manocoreini of the world are keyed. A distribution map of all species is also provided.

## ﻿Introduction

The tribe Manocoreini Hsiao, 1964 is a small group of Coreinae Leach, 1815, which only comprises the genus *Manocoreus* Hsiao, 1964, endemic to China (Li 1996). Hsiao (1964) described *Manocoreus* and four species from southern China as belonging to it: the type species *M.vulgaris* from Fujian, Guangdong, Jiangxi, and Zhejiang; *M.marginatus* from Yunnan; *M.montanus* from Sichuan; and *M.yunnanensis* from Yunnan. Ren (1983) provided brief notes on all four known species of *Manocoreus* and described *M.astinus* from Yunnan. Ren (1993) described *M.grypidus* from Hubei, while Liu and Ren (1993) described *M.furcatus* from Fujian. Up to now, the genus *Manocoreus* contains seven species, and all of which are only found in southern China.

Tribe Manocoreini was established based on characteristics of head and plica on abdominal sternite VII of female, and was considered as closely related to Dasynini Bergroth, 1913 and Gonocerini Mulsant & Rey, 1870 (Hsiao 1964). Li (1996, 1997) published the comparative morphological and cladistic analysis of Coreidae, his result supported Manocoreini as a taxon of tribal rank. Upon examination of Manocoreini collections from China, *Manocoreus* is reviewed, and a new species *Manocoreushsiaoi* sp. nov. is described from Guangxi province, China.

## ﻿Materials and methods

External structures were examined by using a Zeiss Discovery V20 stereomicroscope. Measurements (in mm) were taken using Zeiss ZEN 2.5 pro software.

The male genital capsule and female abdomen were removed in dry condition and soaked in 75 °C, 10% KOH for 30 minutes to one hour to remove muscles. Endosoma was carefully stretched with a pair of forceps under a Zeiss Discovery V20 stereomicroscope. Photographs of habitus and detailed structures were taken by using a Canon EOS 7D Mark II camera equipped with a LAOWA 100 mm F2.8 macro 2× macro lens. Photographs of the genitalic structures were taken using a Canon EOS 7D Mark II camera equipped with a LAOWA 25 mm F2.8 macro 2.5–5× macro lens, or equipped with a tube lens and a Mitutoyo M Plan Apo 10× objective lens. Morphological terminology follows Hsiao (1964), Ren (1993), Brailovsky (2007a, b), Yi and Bu (2015), Zhou and Rédei (2020), and Pluot-Sigwalt and Moulet (2020).

Abbreviations used in the text and figures are as follows:

**aed** aedeagus;

**am** ampulla;

**bp** basal plates;

**cd** coiled duct;

**dpr** dorsoposterior rim;

**ds** duct seminis;

**fz** flexible zone;

**ga** gonangulum;

**lpc** lateroposterior convexes;

**lt8, 9** laterotergites VIII, IX;

**mdp** median projection;

**mvp** median ventroposterior process;

**ph** process on head in front of antenniferous tubercles;

**phth** phallotheca;

**ra8, 9** ramus of valvula VIII, IX;

**rs** ring sclerite;

**s6, 7, 10** sternites VI, VII, X;

**sd** spermathecal duct;

**sp8** spiracle VIII;

**sr** seminal receptacle;

**sth** spermatheca;

**t9, 10** tergites IX, X;

**vcs** ventral conjunctival sclerites;

**va8, 9** valvulae VIII, IX;

**vf8, 9** valvifers VIII, IX.

Label data of type specimens were cited verbatim: a slash (/) separates the lines and a double slash (//) different labels from the same specimen; notes about the label data were indicated in square brackets ([]).

Abbreviations for depositories:

**IZCAS**Institute of Zoology, Chinese Academy of Sciences, Beijing, China;

**NKUM**Institute of Entomology, Nankai University, Tianjin, China;

**SYSBM** Museum of Biology, Sun Yat-sen University, Guangzhou, China.

## ﻿Taxonomy

### ﻿Tribe Manocoreini

Taxon classificationAnimaliaHemipteraCoreidae

Hsiao, 1964

EC4A7FFD-6BB1-5893-8660-594F9E7CE8B8

Manocoreini Hsiao, 1964: 90. Hsiao (1977): 217 (in key), 243 (diagnostic characters); Dolling (2006): 87 (catalogue).

#### Type genus by monotypy.

*Manocoreus* Hsiao, 1964.

### ﻿Genus Manocoreus

Taxon classificationAnimaliaHemipteraCoreidae

Hsiao, 1964

922BEF1F-75E1-5F19-B685-F1C0C432CA67

Figs 1 , 2 , 3 , 4 , 5 , 6 , 7 , 8 , 9 , 10 , 11 , 12 , 13 , 14 , 15 , 16

Manocoreus Hsiao, 1964: 90. Hsiao (1977): 217 (in key), 244 (listed); Ren (1983): 321 (diagnostic characters, habitat, hosted plant); Chen and Li (1999): 128 (catalogue, diagnostic characters); Dolling (2006): 87 (catalogue).

#### Type species by original designation.

*Manocoreusvulgaris* Hsiao, 1964.

#### Diagnosis.

**Figure 1.**
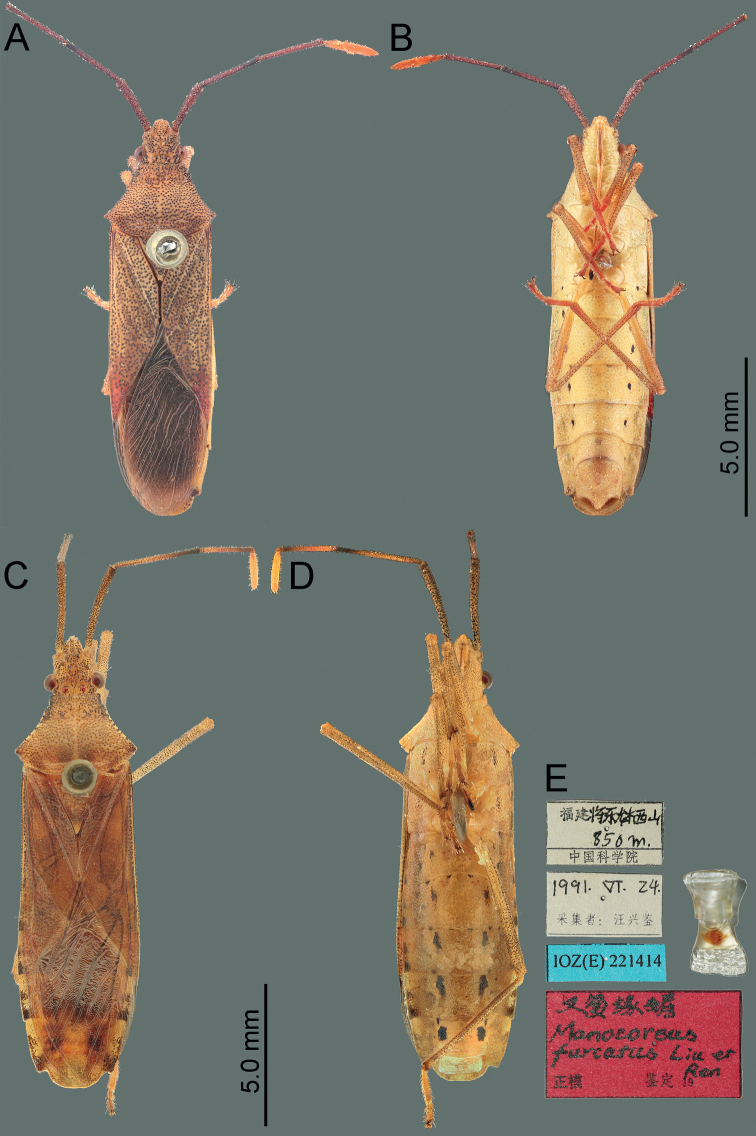
Habitus of *Manocoreus* spp. **A, B***M.astinus* male non-type specimen **A** dorsal view **B** ventral view **C–E***M.furcatus* male holotype **C** dorsal view **D** ventral view **E** labels.

**Figure 2.**
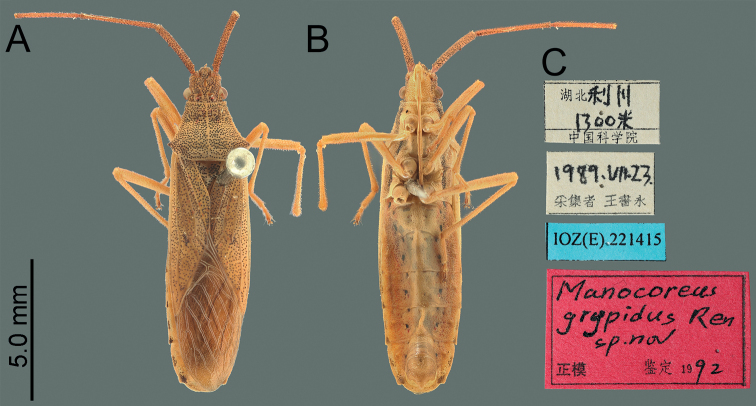
Habitus of *Manocoreusgrypidus* male holotype. **A** dorsal view **B** ventral view **C** labels.

**Figure 3.**
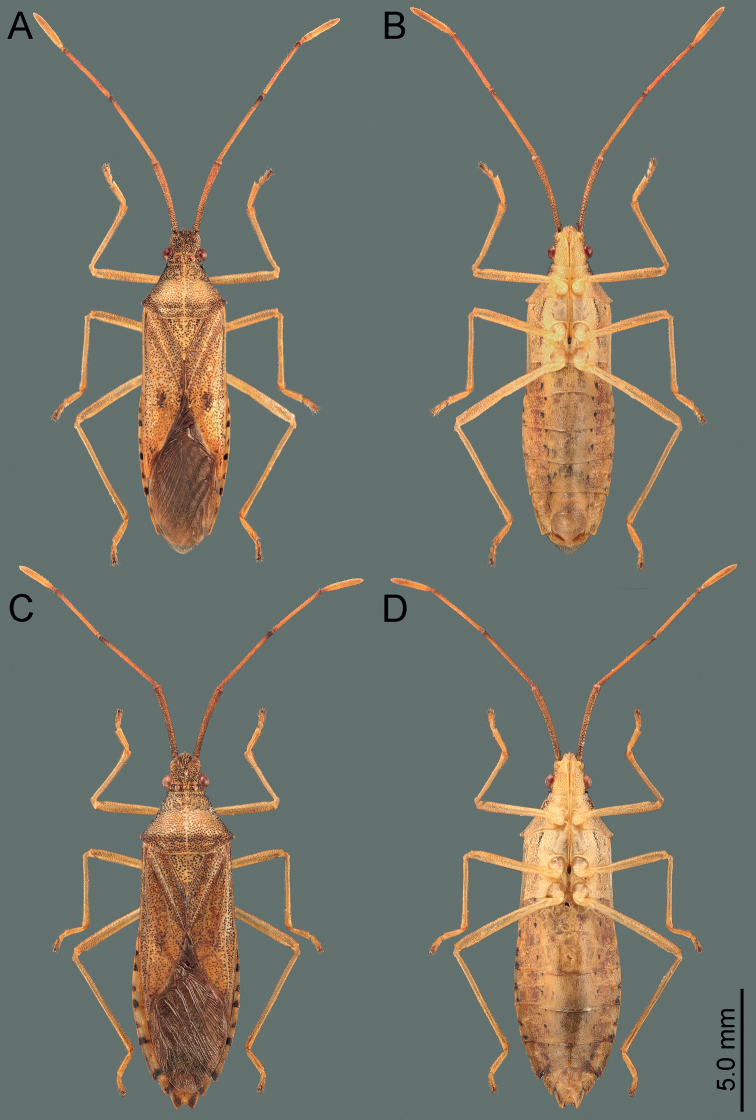
Habitus of *Manocoreushsiaoi* sp. nov. **A, B** male holotype **A** dorsal view **B** ventral view **C, D** female paratype **C** dorsal view **D** ventral view.

**Figure 4.**
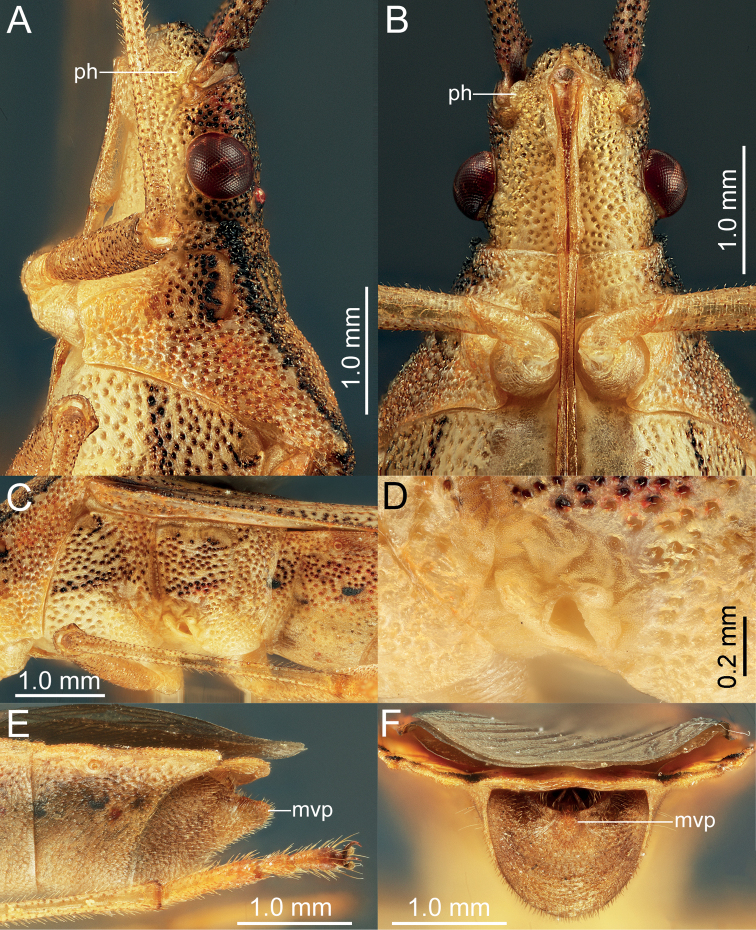
*Manocoreushsiaoi* sp. nov., male holotype **A, B** head and prothorax A lateral view **B** ventral view **C** meso- and metathorax, lateral view **D** scent gland ostiole and peritreme, lateral view **E, F** terminalia **E** lateral view **F** posterior view. Abbreviations: mvp = median ventroposterior process; ph = process on head in front of antenniferous tubercles.

**Figure 5.**
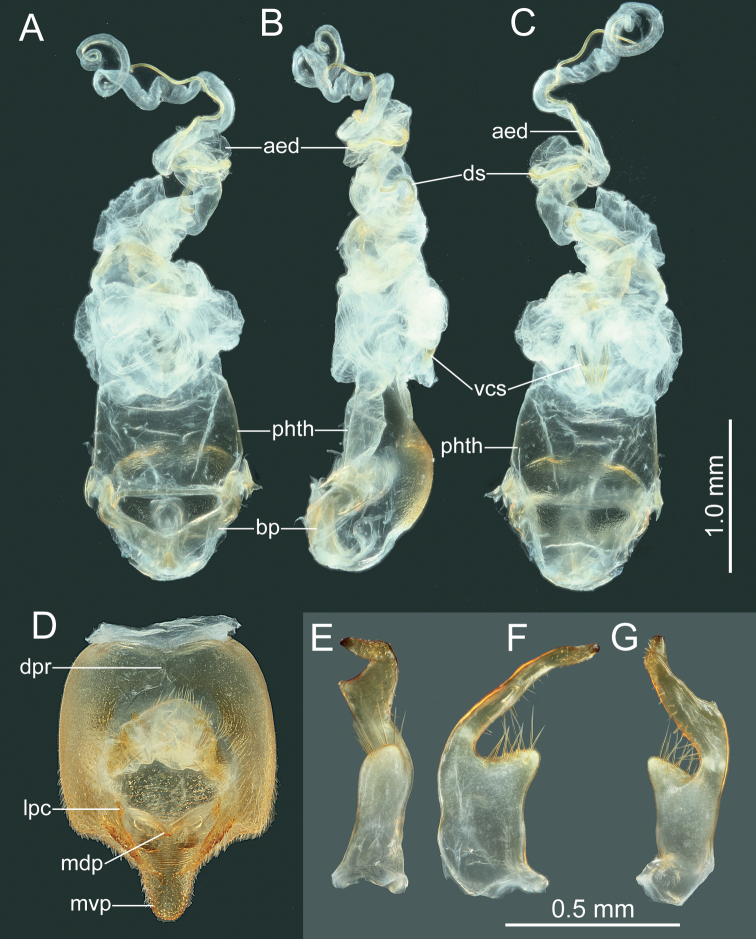
*Manocoreushsiaoi* sp. nov., male genitalia **A–C** phallus **A** dorsal view **B** lateral view **C** ventral view **D** genital capsule, dorsal view **E–G** right paramere in three different aspects. Abbreviations: aed = aedeagus; bp = basal plates; dpr = dorsoposterior rim; lpc = lateroposterior convexes; mdp = median projection; mvp = median ventroposterior process; phth = phallotheca; vcs = ventral conjunctival sclerites.

**Figure 6.**
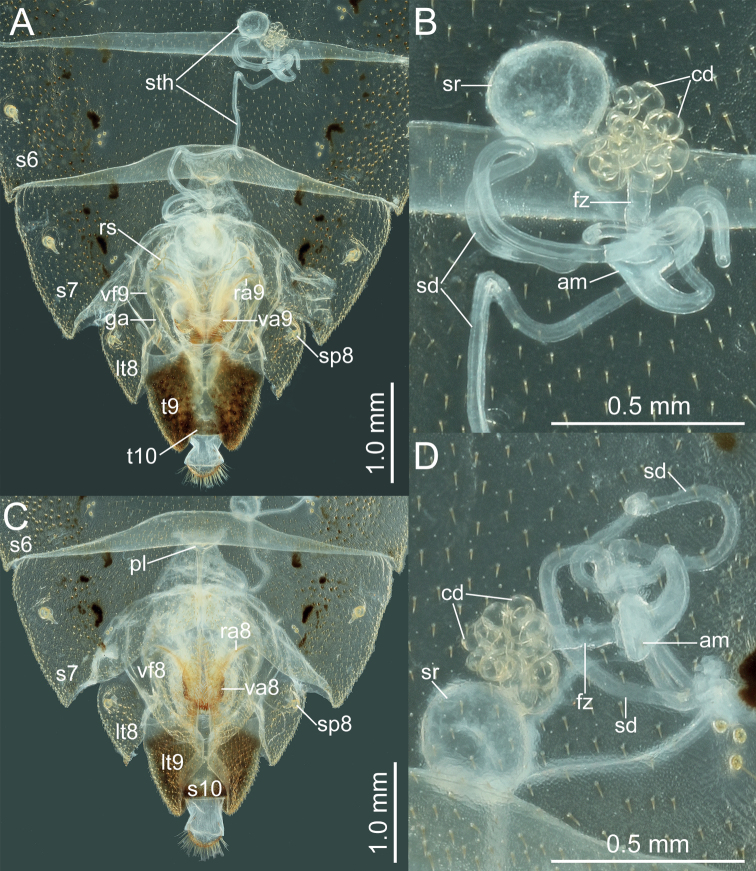
*Manocoreushsiaoi* sp. nov., female genitalia **A, C** terminalia **A** dorsal view **C** ventral view **B, D** spermatheca in two different aspects. Abbreviations: am = ampulla; cd = coiled duct; ds = duct seminist; fz = flexible zone; ga = gonangulum; lt8, 9 = laterotergites VIII, IX; ra8, 9 = ramus of valvula VIII, IX; rs = ring sclerite; s6, 7, 10 = sternites VI, VII, X; sd = spermathecal duct; sp8 = spiracle VIII; sr = seminal receptacle; sth = spermatheca; t9, 10 = tergites IX, X; va8, 9 = valvulae VIII, IX; vf8, 9 = valvifers VIII, IX.

**Figure 7.**
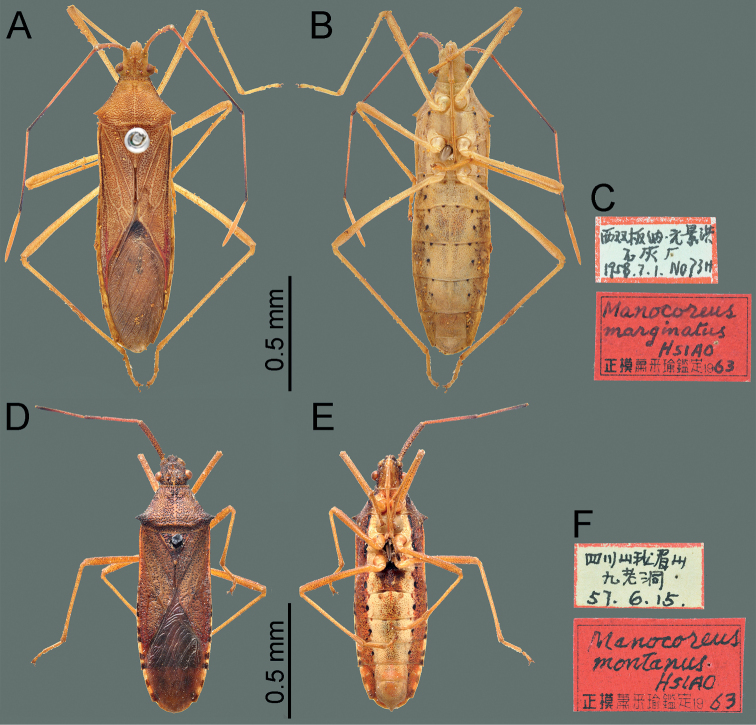
Habitus of *Manocoreus* spp. **A–C***M.marginatus* male holotype **A** dorsal view **B** ventral view **C** labels **D–F***M.montanus* male holotype **D** dorsal view **E** ventral view **F** labels.

**Figure 8.**
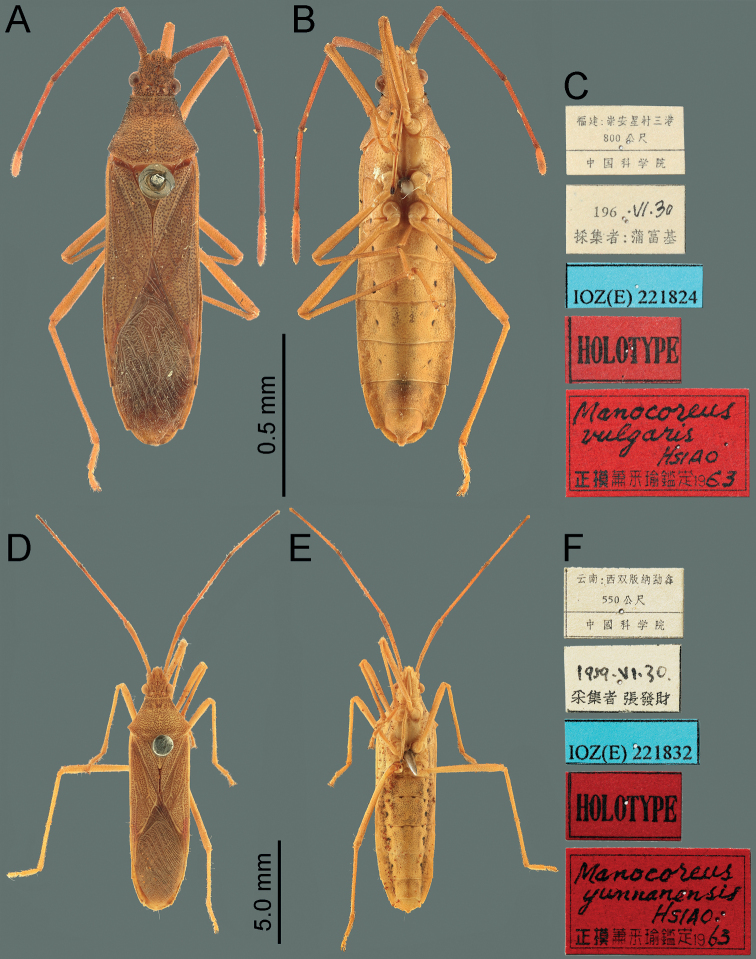
Habitus of *Manocoreus* spp. **A–C***M.vulgaris* male holotype **A** dorsal view **B** ventral view **C** labels **D–F***M.yunnanensis* male holotype **D** dorsal view **E** ventral view **F** labels.

**Figure 9.**
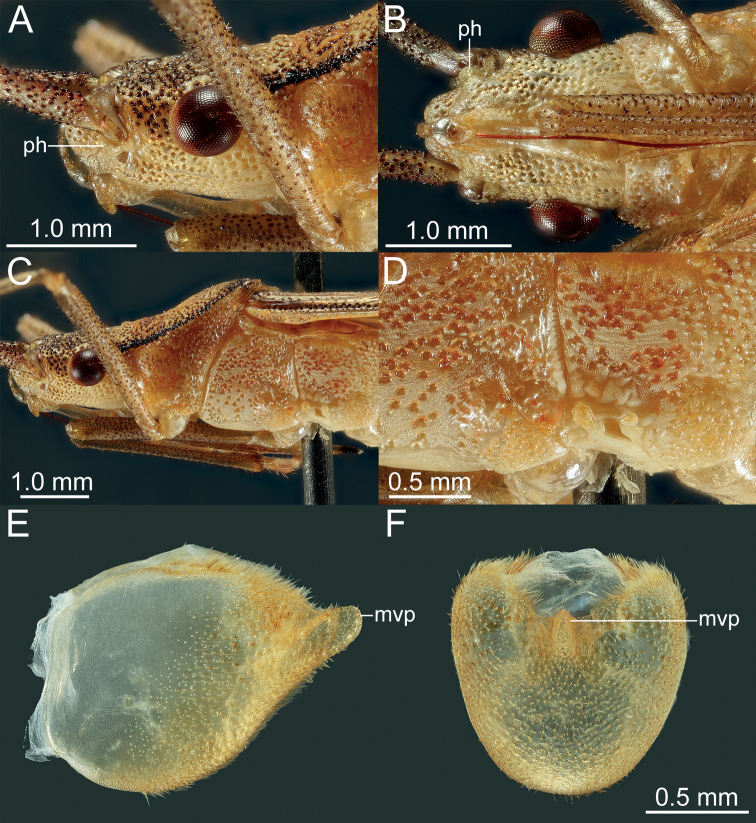
*Manocoreusvulgaris* male non-type specimen **A, B** head **A** lateral view **B** ventral view **C** head and thorax, lateral view **D** scent gland ostiole and peritreme, lateral view **E, F** genital capsule **E** lateral view **F** posterior view. Abbreviations: mvp = median ventroposterior process; ph = process on head in front of antenniferous tubercles.

**Figure 10.**
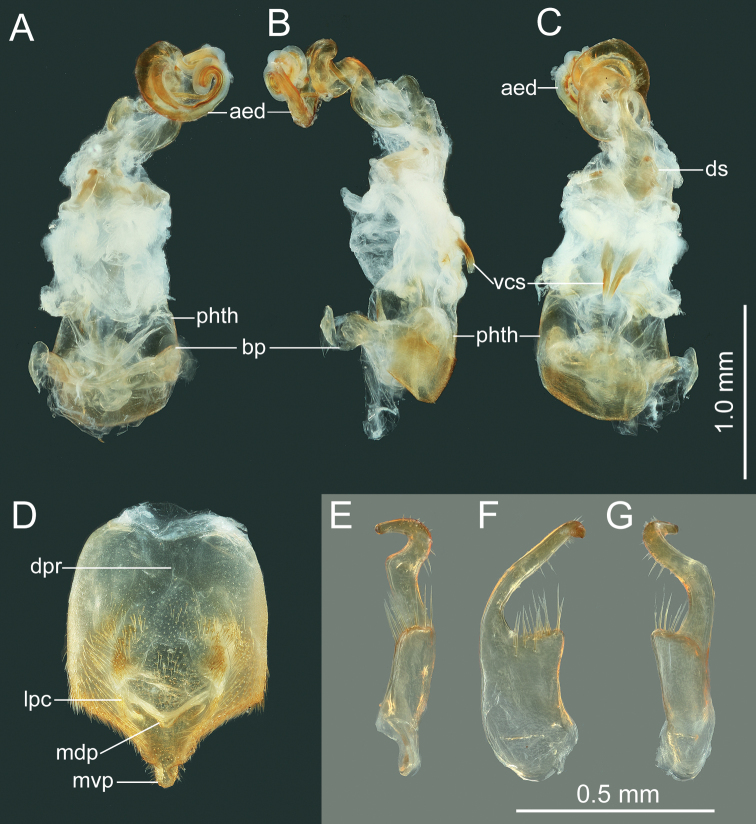
*Manocoreusvulgaris* male non-type specimen, male genitalia **A–C** phallus **A** dorsal view **B** lateral view **C** ventral view **D** genital capsule, dorsal view **E–G** right paramere in three different aspects. Abbreviations: aed = aedeagus; bp = basal plates; dpr = dorsoposterior rim; lpc = lateroposterior convexes; mdp = median projection; mvp = median ventroposterior process; phth = phallotheca; vcs = ventral conjunctival sclerites.

**Figure 11.**
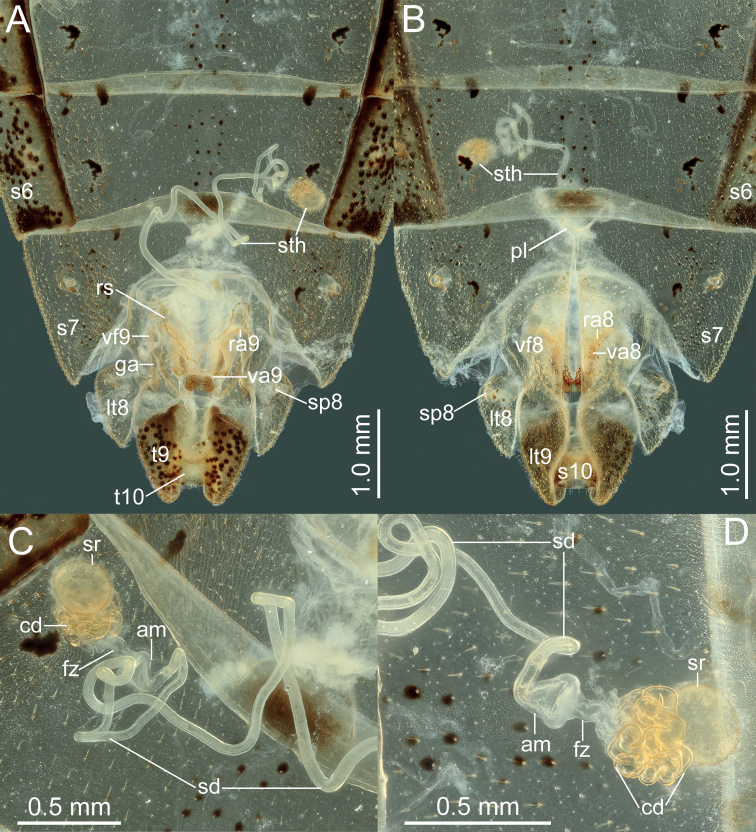
*Manocoreusvulgaris* female non-type specimen, female genitalia **A, C** terminalia **A** dorsal view **B** ventral view **C, D** spermatheca in two different aspects. Abbreviations: am = ampulla; cd = coiled duct; ds = duct seminist; fz = flexible zone; ga = gonangulum; lt8, 9 = laterotergites VIII, IX; ra8, 9 = ramus of valvula VIII, IX; rs = ring sclerite; s6, 7, 10 = sternites VI, VII, X; sd = spermathecal duct; sp8 = spiracle VIII; sr = seminal receptacle; sth = spermatheca; t9, 10 = tergites IX, X; va8, 9 = valvulae VIII, IX; vf8, 9 = valvifers VIII, IX.

**Figure 12.**
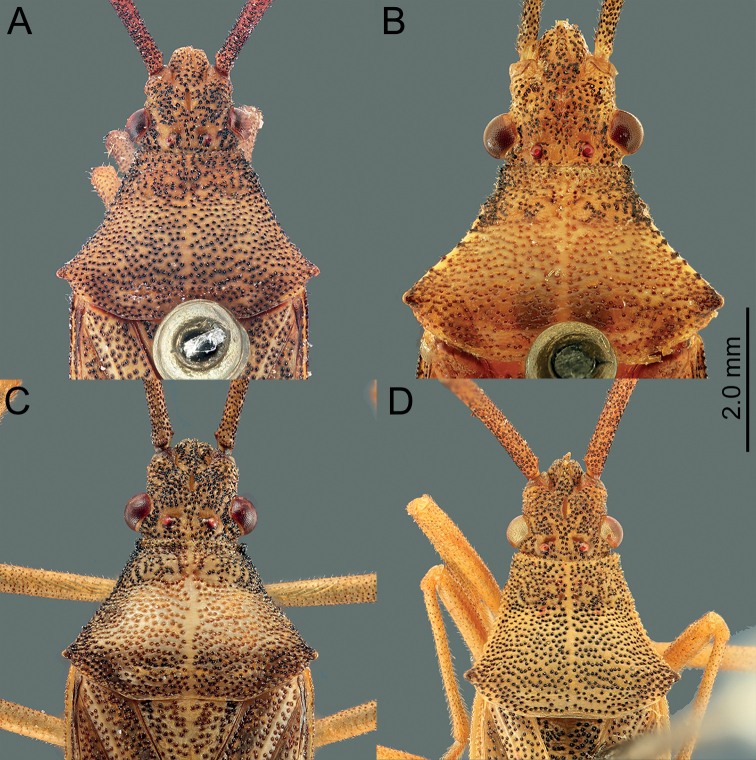
*Manocoreus* spp., head and pronotum, dorsal view **A***M.astinus* (Ren, 1983) **B***M.furcatus* (Liu & Ren, 1993) **C***M.grypidus* (Ren, 1993) **D***M.hsiaoi* sp. nov.

**Figure 13.**
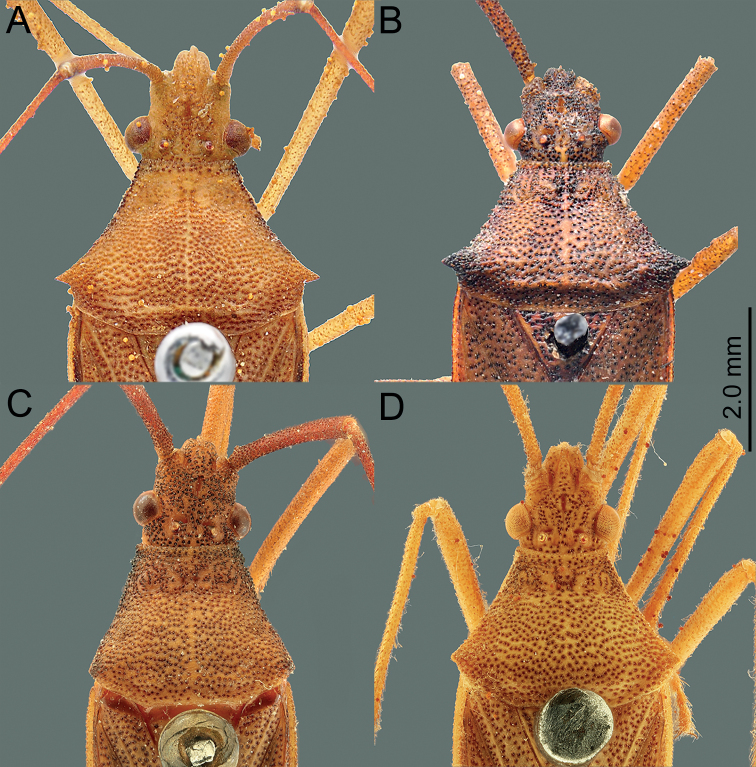
*Manocoreus* spp., head and pronotum, dorsal view **A***M.marginatus* (Hsiao, 1964) **B***M.montanus* (Hsiao, 1964) **C***M.vulgaris* (Hsiao, 1964) **D***M.yunnanensis* (Hsiao, 1964).

**Figure 14.**
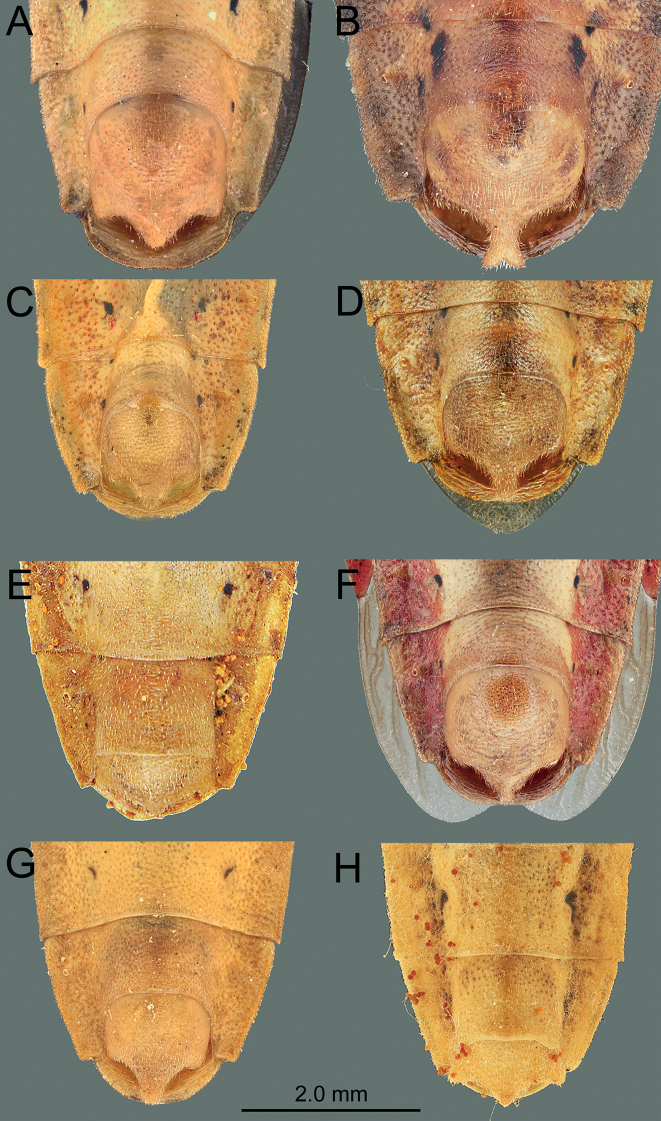
*Manocoreus* spp., male terminalia, ventral view **A***M.astinus* (Ren, 1983) **B***M.furcatus* (Liu & Ren, 1993) **C***M.grypidus* (Ren, 1993) **D***M.hsiaoi* sp. nov. **E***M.marginatus* (Hsiao, 1964) **F***M.montanus* (Hsiao, 1964) **G***M.vulgaris* (Hsiao, 1964) **H***M.yunnanensis* (Hsiao, 1964).

**Figure 15.**
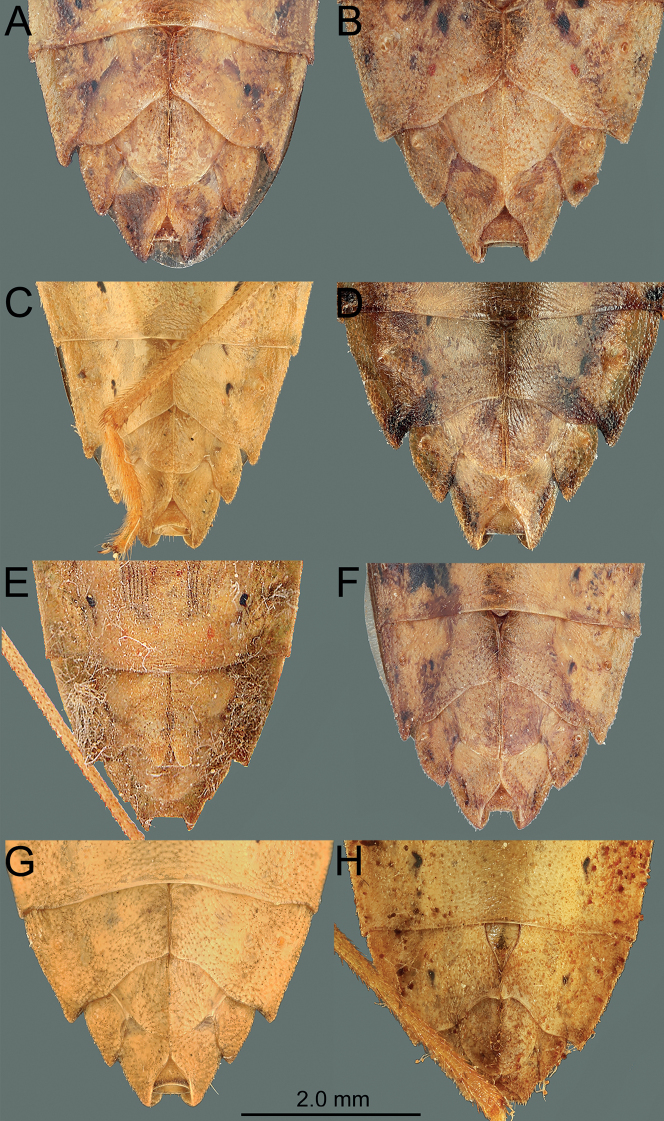
*Manocoreus* spp., female terminalia, in ventral view **A***M.astinus* (Ren, 1983) **B***M.furcatus* (Liu & Ren, 1993) **C***M.grypidus* (Ren, 1993) **D***M.hsiaoi* sp. nov. **E***M.marginatus* (Hsiao, 1964) **F***M.montanus* (Hsiao, 1964) **G***M.vulgaris* (Hsiao, 1964) **H***M.yunnanensis* (Hsiao, 1964).

*Manocoreus* can be distinguished from other genera of Coreinae by the following combined characters: body elongate (Figs 1–3, 7–8); head wide, extending beyond antenniferous tubercles (Figs 12, 13); anteclypeus slender, slightly longer than mandibular plate (Figs 12, 13); head with small dentate or plate-like process in front of antenniferous tubercles (Figs 4A, B, 9A, B: hp); anterior portion of buccula right angled; eye not reaching anterior margin of pronotum (Figs 12, 13); profemora unarmed; vein Cu of hind wing away from base of hamus; meso- and metasternum with a mid-longitudinal groove; dorsum of tibiae sulcate, base of each tibiae slightly protuberant; metatarsal segment I longer than the sum of segments II and III; spiracles of abdomen situated before middle of sternites, near lateral margin (Figs 14, 15); sternum VII of female with middle longitudinal cleft, plica triangular, or rectangular depressed, covered by sternum VI (Figs 6C, 11B, 15); posterior margin of genital capsule broadly sinuate, with median ventroposterior process (Figs 4E, F, 5D, 9E, F, 10D, 14).

#### Redescription.

Body medium to relatively large (11–17 mm), elongate, nearly parallel-sided, ~ 3.2–4.3× as long as humeral width (Figs 12, 13). ***Body surface and vestiture*.** Body surface rather dull; head, thorax, abdomen, with dense punctures; antennae, legs with short, semierect to erect setae, and dense small tubercles; abdomen with short and dense setae.

***Head*** porrect, wider than long, shorter than pronotum, nearly pentagonal, dorsally flat, apex distinctly produced and surpassing antenniferous tubercles; anteclypeus slender, slightly surpassing mandibular plate (Figs 12, 13); antenniferous tubercles almost circular, not prominent; genae with small dentate or plate-like process in front of antenniferous tubercles (Figs 4A, B, 9A, B); anterior of buccula nearly right angled; eyes globular, not reaching anterior margin of pronotum (Figs 12, 13); ocelli not close to each other, relatively close to eyes, dorsally situated before connecting line of posterior edge of eyes (Figs 12, 13); preocellar pit deep, transverse; ocellar tubercles barely raised (Figs 12, 13); antenna four-segmented, antennomere I thickest, apex of antennomere I far from apex of head, with a glabrous and narrow base; antennomeres I–III subcylindrical, subequal in length, antennomere IV fusiform, shortest; labium four-segmented, surpassing posterior margin of mesosternum but not surpassing posterior margin of metasternum (Figs 1B, D, 2B, 3B, D, 7B, E, 8B, E); labial segment I not extending beyond base of head (*M.vulgaris*), or reaching anterior margin of prosternum (other species). ***Thorax*.** Pronotum wider than long, hexagonal, gradually declivent, anterior margin with narrow, indistinct, depressed anterior collar (Figs 12, 13); cicatrices distinct, somewhat depressed; anterolateral margin concave to nearly straight, nodulose; humeral angle rectangular or tapering into stout or acute spine pointing outward (Figs 12, 13); posterolateral angle broadly angulate, posterolateral margin sinuate; posterior margin nearly straight or slight concave; pronotal disk with distinct or indistinct longitudinal medial carinae (Figs 12, 13). Scutellum longer than wide, triangular, apically subacute. Prosternum small, area before mesocoxae of mesosternum large, mesosternum and metasternum with longitudinal median groove; metathoracic scent gland ostiole provided with a bilobate peritreme, evaporatorium extending ~ 1/2 of mesopleuron (Figs 4C, D, 9C, D). Legs unarmed; femora thickened; dorsal surface of tibiae each with a wide longitudinal furrow. Hemelytra macropterous, not reaching or slightly surpassing apex of abdomen (1A, C, 2A, 3A, C, 7A, D, 8A, D); costal margin emarginated, nearly straight, R and M branch from ca. middle of corium, membrane with ~ 10 longitudinal veins; hamus of hind wing very short. ***Pregenital abdomen*.** Connexival segments distinctly raised above tergum, posterolateral angles sometimes acute; sterna without impression or furrow ventrally; spiracles circular, small, close to lateral margin, situated before middle of sterna; spiracle II not visible. Posterior margin of female abdominal tergum VII sinuate, concave in middle, sternum VII of female with middle longitudinal cleft, plica triangular, indistinct, covered by sternum VI (Fig. 14). ***External male genitalia*.** Genital capsule near prolate spheroidal, opening dorsally (Figs 4E, F, 5D, 9E, F, 10D), ventral outline distally concave in lateral view, posterior margin of genital capsule broadly sinuate with median ventroposterior process (Fig. 14); dorsal margin of base of paramere nearly straight, distal portion of paramere sickle-shaped (Figs 5E–G, 10E–G); phallus with a sclerotized articulatory apparatus, phallotheca barrel-shaped, unarmed, conjunctiva rigid and complex, aedeagus coiled, strongly sclerotized (Figs 5A–C, 10A–C). ***External female genitalia*.** Laterotergite VIII subtriangular, spiracles present; laterotergites IX subtriangular, inner margin contact (Fig. 15). Valvifers VIII nearly triangular (Figs 6C, 11B: vf8); valvulae VIII moderately membranous, with posterior distal portions slightly sclerotized (Figs 6C, 11B: va8); gonangulum elongate (Figs 6A, 11A: ga); valvifers IX separated, slender (Figs 6A, 11A: vf9); valvulae IX membranous, with posterior distal portions strongly sclerotized (Figs 6A, 11A: va9); valvulae VIII and IX interlocking through sclerotized rami VIII and IX (Figs 6A, 11A, C); gynatrium with one large ring sclerite (Figs 6A, 11A: rs); spermatheca with a long coiled duct, basal portion of duct slightly expanding, subapical with a conspicuous ampulla (Figs 6B, D, 11C, D: am); distal region with a long flexible zone (Figs 6B, D, 11C, D: fz), tightly coiled duct (Figs 6B, D, 11C, D: cd), and seminal receptacle globose (Figs 6B, D, 11C, D: sr).

#### Distribution.

**Figure 16.**
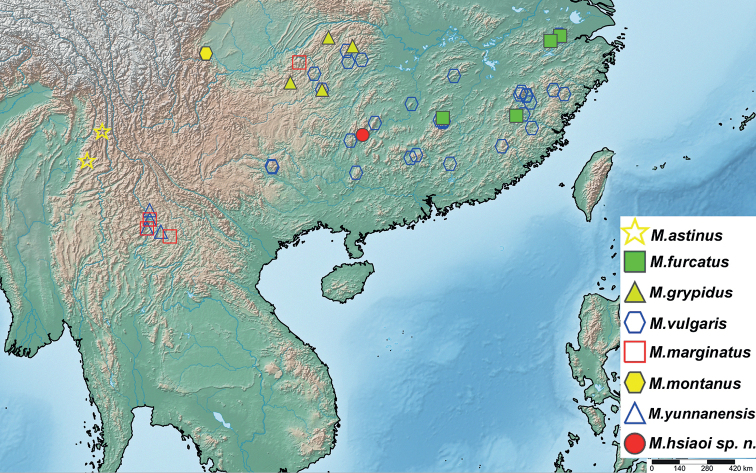
Distribution records of all species of *Manocoreus*.

The genus currently contains eight species, all species distributed in southwestern and southern China (Fig. 16).

### ﻿ Manocoreus astinus

Taxon classificationAnimaliaHemipteraCoreidae

Ren, 1983

CADF453D-1883-584F-AE24-54001BF1C133

Figs 1A, B , 12A , 14A , 15A , 16

Manocoreus astinus Ren, 1983: 322. Holotype: ♂, China, Yunnan, Lushui; IZCAS. Ren (1992): 140 (catalogue, distribution); Dolling (2006): 87 (catalogue, distribution).

#### Material examined.

**China. Yunnan**: Lushui City, Pianma, 2300 m a.s.l., 26.v.1981, leg. S.Y. Wang (1♂ 1♀ NKUM), same but 29.v.1981 (1♂ NKUM), same but 31.v.1981 (1♂ 3♀♀ NKUM); Dehong Prefecture, Yingjiang County, 1300 m a.s.l., 13.iv.1980, leg. S. M. Song (1♂ NKUM).

#### Remarks.

This species is similar to *M.vulgaris* in habitus, size, and color, but differs in the following characters: labium surpassing anterior margin of metacoxae (Fig. 1B); male median ventroposterior process of genital capsule triangular (Fig. 14A).

#### Notes.

The type series of *M.astinus* was not in IZCAS rather than as Ren (1983) stated. Therefore, the authors of this paper examined all specimens which were collected in the type locality with similar dates, and other specimens deposited in NKUM and confirmed their identification as *M.astinus*.

#### Distribution.

**China. Yunnan**: Lushui, Dehong (Fig. 16).

### ﻿ Manocoreus furcatus

Taxon classificationAnimaliaHemipteraCoreidae

Liu & Ren, 1993

9C9EEE22-D58F-5D32-8CD7-78AA5C366E44

Figs 1C–E , 12B , 14B , 15B , 16

Manocoreus furcatus Liu & Ren, 1993: 147. Holotype: ♂, China, Fujian, Jiangle; IZCAS.

#### Type material examined.

***Holotype*** male “Fujian [printed in Chinese] Jiangle [handwritten in Chinese] Longqishan [handwritten in Chinese] / 850 m. [handwritten] / Chinese Academy of Sciences [printed in Chinese] // 1991. VI. 24 [handwritten] / collector: Wang Xingjian [printed in Chinese] // IOZ(E) 221414 [printed] // Chamanyuanchun [handwritten in Chinese] / Manocoreus [handwritten] / furcatus Liu et [handwritten] / Ren [handwritten] / holotype [printed in Chinese] identified [printed in Chinese] 19 [printed]”; IZCAS. ***Paratype*** female, labelled: “Fujian [printed in Chinese] Longqishan [handwritten in Chinese] / 87 [handwritten] year [printed] 7 [handwritten] month [printed] 17 [handwritten] day [printed] / collector [printed in Chinese] Chen Shunli [handwritten in Chinese] // Chamanyuanchun [handwritten in Chinese] / Manocoreus [handwritten] / furcatus Liu et [handwritten] / Ren [handwritten] / paratype [printed in Chinese]”; NKUM.

#### Other material examined.

**China. Jiangxi**: Jinggangshan Xiaoxidong Forest Farm, 24.vii.2002, leg. H.J. Xue (1♂ NKUM); **Zhejiang**: Lin’an volcano Dashi Valley, 400–750 m a.s.l., 9.viii.2007, leg. W. B. Zhu (1♂ 1♀ NKUM), Lin’an Qingliangfeng Botanical Garden, 900–990 m a.s.l., 13.viii.2007, leg. G. P. Zhu (3♂♂ NKUM).

#### Remarks.

*Manocoreusfurcatus* can be recognized from all other species of *Manocoreus* by the following characteristics: distinctly bigger size (Fig. 1C, D, body length 16.0–17.2 mm); middle of both sides of sterna III to VII with large irregular black spots (Fig. 1D); median ventroposterior process of genital capsule bifurcate (Fig. 14B).

#### Distribution.

**China. Fujian**: Jiangle; **Jiangxi**: Jianggangshan; **Zhejiang**: Lin’an (Fig. 16).

### ﻿ Manocoreus grypidus

Taxon classificationAnimaliaHemipteraCoreidae

Ren, 1993

A8C999A1-15E9-54FE-A05B-5825DB01A3B1

Figs 2A–C , 12C , 14C , 15C , 16

Manocoreus grypidus Ren, 1983: 347. Holotype: ♂, China, Hubei, Lichuan; IZCAS. Ren and Xiong (1993): 189 (catalogue, distribution).

#### Type material examined.

***Holotype*** male “Hubei [printed in Chinese] Lichuan [handwritten in Chinese] / 1300 m [handwritten in Chinese] / Chinese Academy of Sciences [printed in Chinese] // 1989.VII.23 [handwritten in Chinese] / collector Wang Shuyong [printed in Chinese] // IOZ(E) 221415 [printed] // Manocoreus [handwritten] / grypidus Ren [handwritten] / holotype [printed in Chinese] identified [printed in Chinese] 19 [printed] 92 [handwritten in Chinese]”; IZCAS.

#### Other material examined.

**China. Guizhou**: Zunyi City Suiyang County Kuankuangshui National Nature Reserve Rangshui, 900 m a.s.l., 13.viii.2010, leg. X. Sun, Y.H. Wang (2♂♂ 5♀♀ NKUM); Fanjingshan Tongkuangchang, 700 m a.s.l., 28.vii.2001, leg. W.B. Zhu (2♀♀ NKUM), same but leg. W.J. Bu (1♂ NKUM).

#### Remarks.

This species can be recognized from all other species of *Manocoreus* by the following characteristics: pronotum with dense black punctures, but lateral margin not black (Fig. 12D); the middle portion of corium with small black spot (Fig. 2A); male median ventroposterior process of genital capsule triangular in ventral view, lateral processes on posterior margin of genital capsule smaller than median process, directed backward and slightly inward in ventral view (Fig. 14C).

#### Distribution.

**China. Guizhou**: Tongren, Zunyi; **Hubei**: Hefeng (Ren 1993), Lichuan (Fig. 16).

### ﻿ Manocoreus hsiaoi sp. nov.

Taxon classificationAnimaliaHemipteraCoreidae

8D144DA0-CAEC-583A-860E-6DCAF30BB6F3

https://zoobank.org/00EB1E86-DA91-4E1C-91BE-20846F1C02D8

Figs 3 , 4 , 5 , 6 , 12D , 14D , 15D , 16

#### Type material.

***Holotype*** (♂) **China: Guangxi Province**, Xing’an County, Maoershan: 900–1320 m a.s.l., 2009-VII-10, leg. Zhong-Hua FAN, mounted on card (NKUM). ***Paratypes*** (1♂, 1♀) same data as holotype, mounted on cards (NKUM); (5♂♂, 3♀♀) **China: Guangxi Province**, Xing’an County, Maoershan: 900–1320 m a.s.l., 2009-VII-10, leg. Qing ZHAO, mounted on cards (NKUM); (1♂) **China: Guangxi Province**, Xing’an County, Maoershan: 900–1320 m a.s.l., 2009-VII-10, leg. Xi SUN, mounted on cards (NKUM).

#### Diagnosis.

*Manocoreushsiaoi* sp. nov. can be recognized from all other species of *Manocoreus* by the following characters: lateral margin of pronotum black (Figs 4A, 12D); punctures on the discal region of pronotum not black (Fig. 12D); connexivum with black spots (Fig. 3A, C); the middle portion of corium with large black spots (Fig. 3A, C); male median ventroposterior process of genital capsule long and tuberculate (Figs 4E, 5D, 14D).

#### Description.

Body elongate, ~ 3.76–3.94× as long as humeral width (Fig. 3A–D). ***Color*, *integument*, *and vestiture*.** Body brownish yellow, dorsum of head with dense black punctures, underside of head with dense punctures concolorous with body surface (Fig. 4B), compound eyes dark red, ocelli reddish; labium yellow, distal one fourth of segment IV black; antennomeres I–III brownish yellow, antennomere IV paler, apical portion of antennomeres II and III blackish, antennomere I with moderately dense small black tubercles, small tubercles on antennomeres II and III more scattered and paler, each segments with short semi-erect setae; lateral margin of pronotum black (Fig. 4A); collar, callus area, area near lateral margin and posterior margin with black punctures, central discal region of pronotum with yellow punctures; propleura and prosternum yellow, propleura with black punctures (Fig. 4C); scutellum and corium brownish yellow, with brown to black punctures, meso- and metapleura, and meso- and metasternal yellow, meso- and metapleura with black punctures; legs yellow, only apical area of tarsal segment III blackish brown, femora and tibiae of each leg with moderately dense small brownish tubercles, femora, tibiae and tarsi of each leg with short semi-erect setae, setae on apical half of tibiae and tarsi denser; corium with a large and more or less central black spot, membrane grey, basal area darker; middle and apical portion of connexivum of each segment black, venter of abdomen yellowish brown, abdominal terga with a median black spot on each side of segments II–VII; a pair of smaller black spots located near anterior margin of segments III to VII (Fig. 3).

#### Structures.

***Head*.** Width of head ~ 1.07–1.26× as wide as median length of head, ~ 1.71–1.80 time as wide as interocular distance; antennomere I slightly shorter than antennomere II, ratio of antennomeres I:II:III:IV = 1:1.03:0.84:0.62; apex of labium surpassing posterior margin of mesocoxae, not reaching to anterior margin of metacoxae. ***Pronotum*** ~ 1.43–1.60× as width across humeral angles as its median length; scutellum ~ 0.96–1.16× as long as its width. Anterior peritreme of metathoracic scent gland slightly larger than protruding posterior peritreme, gyrification of evaporatorium deep (Fig. 4C, D). ***Pregenital abdomen*.** Abdomen oblong, spiracles situated near lateral margin of abdominal sterna III–VII (segments III to VIII in female), before middle line of each segment; sternum VII of male strongly concave medially, length of concave part ~ 1/2 length of sternum VIII in ventral view; in female plica triangular, and partly exposed out of sternum VI, posterior margin of sternum VII sinuated. ***External male genitalia*.** Genital capsule opening dorsally (Fig. 4E, F), dorsoposterior rim wide, median projection conspicuous, triangular (Fig. 5D: mdp), posterior margin of genital capsule broadly sinuate and ventroposterior process median (Fig. 5D: mvp), lateral portion of posterior margin roundly produced on each side (Fig. 5D); dorsal margin of base of paramere near straight, ventral region of base of paramere with triangular lobe (Fig. 5E–G), distal portion sickle-shaped (Fig. 5E–G); phallus with a sclerotized articulatory apparatus (Fig. 5A–C), phallotheca barrel-shaped, unarmed (Fig. 5A–C: phth), conjunctiva rigid and complex, with one pair of membranous processes and a pair of slender, triangular adjacent ventral conjunctival sclerites (Fig. 5B, C: vcs), aedeagus coiled, strongly sclerotized, distal portion tubular, obliquely truncate apically (Fig. 5A–C: aed). ***External female genitalia*.** Laterotergite VIII subtriangular, with sinuate inner margin, spiracles nearer to basal margin than to lateral margin; laterotergites IX subtriangular, posterior portion of inner margin concave (Figs 6C, 14D). Valvifers VIII nearly triangular, posterior portion of outer margin slightly convex (Fig. 6C: vf8); posterior distal portions of valvulae VIII with dense hair-like setae (Fig. 6C: va8); valvifers IX slightly sclerotized (Fig. 6A: vf9); distal portions of valvulae IX strongly sclerotized, sheath-like, downcurved (Fig. 6A: va9); ring sclerite of gynatrium large, slender and curved (Fig. 6A: rs); spermatheca with a conspicuous ampulla subapically, distal area of ampulla expanded, body of ampulla elongate (Fig. 6B, D: am); distal region with relatively long flexible zone (Fig. 6B, D: fz), with sclerotized, tightly tangled coiled duct (Fig. 6B, D: cd), and not distinctly sclerotized seminal receptacle globose (Fig. 6B, D: sr).

#### Measurements

**(in mm).** Male holotype / male paratypes (*n* = 5) / female paratypes (*n* = 4): total body length 13.57 / 12.02–13.64 / 13.65–14.95; head length 1.50 / 1.53–1.66 / 1.55–1.74, maximum width of head across eyes 1.89 / 1.79–1.96 / 1.83–1.99, interocular distance 1.10 / 1.03–1.11 / 1.07–1.11, preocular distance 0.82 / 0.82–0.92 / 0.83–1.00, postocular distance 0.14 / 0.13–0.21 / 0.12–0.22, interocellar distance 0.51 / 0.42–0.53 / 0.51–0.52; length of antennomere I 3.18 / 2.82–3.26 / 2.96–3.16, II 3.28 / 3.00–3.33 / 3.08–3.21, III 2.68 / 2.43–2.70 / 2.44–2.58, IV 1.89 / 1.90–2.02 / 1.74–1.83; pronotum middle length 2.25 / 2.05–2.51 / 2.34–2.53, maximum width across frontal angles 1.57 / 1.43–1.59 / 1.52–1.66, maximum width across humeral angles 3.59 / 3.16–3.58 / 3.58–3.92; scutellum length 1.76 / 1.50–1.58 / 1.60–1.81, scutellum width 1.52 / 1.30–1.58 / 1.52–1.59

#### Etymology.

This specific epithet is dedicated to the memory of Prof. Hsiao Tsai-Yu (1903–1978), founder of the modern Heteroptera research in China (Zheng et al. 1979).

#### Distribution.

**China. Guangxi**: Xing’an (Fig. 16).

### ﻿ Manocoreus marginatus

Taxon classificationAnimaliaHemipteraCoreidae

Hsiao, 1964

8DAC5098-D0AE-5AC3-9631-335E2DD684A7

Figs 7A–C , 13A , 14E , 15E , 16

Manocoreus marginatus Hsiao, 1964: 92. Holotype: ♂, China, Yunnan, Jinghong; IZCAS. Hsiao (1977): 244 (description, in key, photo), 245 (description, distribution); Ren (1983): 321 (in key), 323, 324 (figs); Zhu and Bu (2005): 182 (catalogue, distribution, description).

#### Type material examined.

***Holotype*** male labelled: “Xishuangbanna • Yuanjinghong [handwritten in Chinese] / Shihuichang [handwritten in Chinese] /1958.7.1 NO73H [handwritten] // Manocoreus [handwritten] / marginatus [handwritten] / HSIAO [handwritten] / holotype [printed] Hsiao Tsaiyu identified [printed in Chinese] 19 [printed] 63 [handwritten in Chinese]”; NKUM. ***Allotype*** female, labelled: “Xishuangbanna • Damenglong [handwritten in Chinese] / No. 94H [handwritten] /195-VIII-4 [handwritten] // Manocoreus [handwritten] / marginatus [handwritten] / HSIAO [handwritten] / allotype [printed] Hsiao Tsaiyu identified [printed in Chinese] 19 [printed] 63 [handwritten in Chinese]”; NKUM.

#### Other material examined.

**China. Yunnan**: Xishuangbanna Damenglong, 650 m a.s.l., 6.v.1958, leg. C.P. Hong (1♀ IZCAS), Mengla County Shangyong town, 1–3.viii.2007, leg. L. Shi (1♀ SYSBM).

#### Remarks.

This species is similar to *M.yunnanensis* in habitus, size, and color, but differs in the following characters: lateral margin of pronotum black (Fig. 13A); subcostal margin of forewing black (Fig. 7A); distal portion of median ventroposterior process of the genital capsule with a round upward hook-shaped process in lateral view (see Ren 1983: fig. 29); middle of female sternum VII sharply concave, both sides with round process backward (Fig. 15E).

#### Distribution.

**China. Guizhou**: Daozhen (Zhu and Bu 2005); **Yunnan**: Xishuangbanna (Fig. 16).

### ﻿ Manocoreus montanus

Taxon classificationAnimaliaHemipteraCoreidae

Hsiao, 1964

107990FF-195A-535B-94A9-82162CBDEB91

Figs 7D–F , 13B , 14F , 15F , 16

Manocoreus montanus Hsiao, 1964: 90. Holotype: ♂, China, Sichuan, Mount Emei; NKUM. Hsiao (1977): 244 (description, distribution, in key, photo); Ren (1983): 321 (in key), 323, 324 (figures).

#### Type material examined.

***Holotype*** male labelled: “Sichuan Emeishan [handwritten in Chinese] / Jiulaodong [handwritten in Chinese] /57.6.15 [handwritten] // Manocoreus [handwritten] / montanus [handwritten] / HSIAO [handwritten] / holotype [printed] Hsiao Tsaiyu identified [printed in Chinese] 19 [printed] 63 [handwritten in Chinese]”; NKUM. ***Allotype*** female, labelled: “Sichuan Emeishan [printed in Chinese] / Jiulaodong [printed in Chinese] 1800 m [printed] / 1957.7.8 [handwritten] /Zheng Leyi • Cheng Hanhua [printed in Chinese] // Manocoreus [handwritten] / marginatus [handwritten] / HSIAO [handwritten] / allotype [printed] Hsiao Tsaiyu identified [printed in Chinese] 19 [printed] 63 [handwritten in Chinese]”; NKUM.

#### Other material examined.

**China. Sichuan**: Emeishan Jiulaodong 1800–1900 m a.s.l., 9.vii.1957, leg. F.X. Zhu (1♂ IZCAS), same but 17.viii.1957, leg. Z.Y. Wang (1♀ IZCAS), same but 19.viii.1957, leg. Z.Y. Wang (1♂ IZCAS), Chudian, 1783 m a.s.l., 28.vi.1957, leg. Z.Y. Wang (1♂ IZCAS), Xixiangchi, 1800–2000 m a.s.l., 30.viii.1957, leg. Y.C. Lu (1♀ IZCAS).

#### Remarks.

This species can be recognized from all other species of *Manocoreus* by the following characteristics: antennomere III dilated apically; lateroventral side of the head, thorax, and abdomen with wide, dark reddish to brownish longitudinal stripe (Fig. 7E); lateral process on posterior margin of genital capsule round (Fig. 14F).

#### Distribution.

**China. Sichuan**: Emeishan (Fig. 16).

### ﻿ Manocoreus vulgaris

Taxon classificationAnimaliaHemipteraCoreidae

Hsiao, 1964

673D44B0-C627-5AAD-A1D3-811B976965F6

Figs 8A–C , 9 , 10 , 11 , 13C , 14G , 15G , 16

Manocoreus vulgaris Hsiao, 1964: 91. Holotype: ♂, China, Fujian, Chong’an; IZCAS. Hsiao (1977): 244 (description, distribution, in key, photo); Ren (1983): 321 (in key), 323, 324 (figures); Chen et al. (1993): 411 (catalogue, distribution); Ren and Xiong (1993): 189 (catalogue, distribution); Bu et al. (1995): 128 (catalogue, distribution); Liu (1998): 89 (catalogue, distribution); Chen and Li (1999): 128 (catalogue, distribution, description, host plant); Bu and Zheng (2001): 276 (catalogue, distribution); Zhu and Bu (2006): 239 (catalogue, distribution, description);
Manocoreus valgaris : Ren (1983): 321, 323, 324 (in key, figures) [incorrect subsequent spelling].

#### Type material examined.

***Holotype*** male labelled: “Fujian: Chong’an Xingcun Sangang [printed in Chinese] / 800 [printed] Gongchi [printed in Chinese] / Chinese Academy of Sciences [printed in Chinese] // 196 [printed] •VI•30 [handwritten] / collector: Pu Fuji [printed in Chinese] // IOZ(E) 221824 [printed] // ***Holotype*** [printed] // Manocoreus [handwritten] / vulgaris [handwritten] / HSIAO [handwritten] / holotype Hsiao Tsaiyu identified [printed in Chinese] 19 [printed] 63 [handwritten]”; IZCAS. ***Paratype*** male, labelled: “Fujian: Chong’an Xingcun Sangang [printed in Chinese] / 720–800 [printed] Gongchi [printed in Chinese] / Chinese Academy of Sciences [printed in Chinese] // 196 [printed] 0•VI•30 [handwritten] / collector: Jiang Qiaoyun [printed in Chinese] // ***Paratype*** [printed] / Manocoreus [handwritten] / vulgaris [handwritten] / HSIAO [handwritten]”; NKUM.

#### Other material examined.

**China. Fujian**: Chong’an Xingcun Sangang, 900–1000 m a.s.l., 7.vii.1960, leg. Y.R. Zhang (1♂ IZCAS), same but 740–910 m a.s.l., 25.v.1960, leg. C.L. Ma (1♂ IZCAS), Chong’an Xingcun Tongmuguan, 850–970 m a.s.l., 8.vii.1960, leg. Y.R. Zhang (1♂ IZCAS), same but 900–1150 m a.s.l., 10.vii.1960, leg. C.L. Ma (1♂ IZCAS), Chong’an Xingcun Guadun, 950–1210 m a.s.l., 12.vi.1960, leg. Y. Zuo (1♀ IZCAS), same but 840–1210 m a.s.l., 21.vi.1960, leg. Y.R. Zhang (1♂ IZCAS), same but 900–1160 m a.s.l., 8.vii.1960, leg. C.L. Ma (1♂ IZCAS), Meihuashan Sanhuicun, 3.v.2004, leg. C.X. Yuan, J. Li (1♂ NKUM); **Guangdong**: Lianzhou City Dadongshan Nature Reserve, 18.vii.2004, leg. X.M. Li (1♀ NKUM), Lianzhou Dadongshan, 13–16.vi.2007, leg. L.L. Huang (1♀ SYSBM), Lianxian [= Lianzhou City] Dadongshan, 30.vii.2007, leg. H.D. Chen (1♀ SYSBM), same but leg. Z.Y. Chen (1♂ SYSBM), same but 3–9.vii.2008, leg. H.D. Chen (1♂ 2♀♀ SYSBM), same but 3–9.vii.2008 leg. Z.Y. Chen (1♂ 2♀♀ SYSBM), same but 3–6.viii.2010, leg. H.D. Chen (2♀♀ SYSBM), same but 3–6.viii.2010, leg. Z.Y. Chen (1♂ 6♀♀ SYSBM), same but 3–6.viii.2010, leg. Z.Y. Chen (1♂ 6♀♀ SYSBM), same but 3–6.viii.2010, leg. W.C. Xie (1♂ 1♀ SYSBM), Nanling Dadongshan, 24.vi.2009, leg. D.D. Fang (1♂ SYSBM), same but 25.vi.2009, leg. X.L. Han (1♂ 2♀♀ SYSBM), same but 22.vi.2009, leg. F.L. Jia (1♂SYSBM); **Guangxi**: Jinxiu Dayaoshan Nature Reserve Shengtangshan Protection Station, 780–1200 m a.s.l., 22.vii.2009, leg. Z.H. Fan (1♂ 1♀ NKUM), same but leg. Q. Zhao (1♂ NKUM), same but 1200 m a.s.l., 23.vii.2009, leg. X. Sun (1♀ NKUM), same but 1200 m a.s.l., 23.vii.2009, leg. K. Dang (1♀ NKUM), Jinxiu Dayaoshan Nature Reserve Yinshan Station, 1150 m a.s.l., 25.vii.2009, leg. K. Dang (1♀ NKUM), same but leg. Z.H. Fan (1♂ NKUM), Longsheng Huaping Nature Reserve, 800–1280 m a.s.l., 17.vii.2009, leg. X. Sun (1♀ NKUM), Tianlin Langping Linaoshan, 1400 m a.s.l., 28.v.2002, leg. G.F. Jiang (1♀ NKUM), Tianlin Laoshanlinchang, 1.vi.2002, leg. X.J. Yang (1♂ NKUM); **Guizhou**: Yanhe County Mayanghe Nature Reserve Huangtu township, 607 m a.s.l., 29.vii.2014, leg. X.J. Peng et al. (2♂♂ 2♀♀ IZCAS); **Hunan**: Yizhang Mangshan 1100–1270 m a.s.l., 22.vii.2004, leg. W.B. Zhu (1♂ 4♀♀ NKUM), same but leg. J.L. Li (2♂♂ NKUM), same but leg. J.Y. Xu (2♂♂ 1♀ NKUM), same but 1050–1300 m a.s.l., 23.vii.2004, leg. W.B. Zhu (1♀ NKUM), Yizhang County Mangshan Nature Reserve Xuzichong, 487 m a.s.l., 22.viii.2014, leg. H.Q. Yin et al. (2♂♂ IZCAS), Yizhang County Mangshan Nature Reserve Yiping, 750 m a.s.l., 18.viii.2014, leg. H.Q. Yin et al. (1♀ IZCAS), Yanling County Taoyuandong, 660–800 m a.s.l., 16.vii.2004, leg. W.B. Zhu (1♂ NKUM), same but leg. Y.L. Ke (1♀ NKUM), same but 1000 m a.s.l., 26.vii.2004, leg. J.L. Li (1♂ NKUM), Hengyang Hengshan, 1030 m a.s.l., 20.vii.2004, leg. J.Y. Xu (1♂ 1♀ NKUM), same but leg. J.L. Li (1♂ NKUM), same but leg. Y. Tian (1♂ NKUM), same but 335–610 m a.s.l., leg. J.Y. Xu (1♂ NKUM), same but leg. J.L. Li (1♀ NKUM), Hengyang Hengshan Tianzhufeng, 1030 m a.s.l., 26.vii.2004, leg. W.B. Zhu (1♂ NKUM), Dong’an County Shunhuangshan, 470–900 m a.s.l., 27.vii.2004, leg. J.Y. Xu (1♂ NKUM), Sangzhi County Badagongshan Xiaozhuangping, 1420 m a.s.l., 18.vi.2015, leg. H.B. Liang (1♂ IZCAS), Zhangjiajie City Wulingyuanqu Magongting, 700 m a.s.l., 9.vi.2015, leg. H.B. Liang (1♂ IZCAS); **Jiangxi**: Yifeng Yuanqian, 15.v.1959, (1♂ IZCAS), same but 19.v.1959, (1♀ IZCAS), same but 15.vi.1959, (1♀ IZCAS), same but 21.vi.1959, (1♂ IZCAS), Jinggangshan Zaohemu, 22.vii.2002, leg. H.J. Xue (2♂♂ 1♀ NKUM), Jinggangshan Xiaoxidong, 24.vii.2002, leg. H.J. Xue (1♂ NKUM), Jinggangshan Wuzhifeng Xiaoxidong, 24.vii.2002, leg. X. Yu (1♀ NKUM), Jinggangshan Dabali Forest Farm, 26.vii.2002, leg. J.H. Ding (1♀ NKUM), Jiulianshan Xiagongtang, 5.vii.2002, leg. W.L. Zhang, J.H. Ding (2♂♂ NKUM), Jiulianshan Xiagongtang Pingkeng, 14–15.vii.2002, leg. X. Yu (1♀ NKUM), Jiulianshan Pingkeng, 16.vii.2002, leg. H.J. Xue (1♀ NKUM); **Zhejiang**: Lin’an Tianmushan, 300–700 m a.s.l., 8.viii.2007, leg. W.B. Zhu (4♂♂ 5♀♀ NKUM), same but leg. Z.H. Fan (2♂♂ 2♀♀ NKUM), Lin’an Huoshan Dashigu, 400–750 m a.s.l., 9.viii.2007, leg. W.B. Zhu (1♂ 2♀♀ NKUM), Wuyanling, 700 m a.s.l., 3.viii.2007, leg. G.P. Zhu (1♂ 1♀ NKUM).

#### Remarks.

This species can be recognized from all other species of *Manocoreus* by the following characteristics: humeral angles of pronotum rectangular or slightly acute (Fig. 13C); lateral side of head and thorax without black spots or stripes (Figs 8B, 9A–D); median ventroposterior process of genital capsule subtriangular in ventral view (Fig. 14G), lateral processes on posterior margin of genital capsule round in ventral view (Fig. 14G). Based on the examination of a series of specimens distributed from seven provinces of southern China, the morphological characteristics of *Manocoreusvulgaris* has a moderate degree of geographical variation in body size, coloration, and shape of spine of humeral angles, and all of them are considered in intraspecific range.

#### Distribution.

**China. Fujian**: Chong’an, Jianyang (Chen et al. 1993, Chen and Li 1999), Longyan, Meihuashan, Shunchang (Chen and Li 1999), Wuyishan (Chen and Li 1999), Youxi (Chen and Li 1999); **Guangdong**: Lianzhou; **Guangxi**: Jinxiu, Longsheng, Tianlin; **Guizhou**: Fanjingshan (Zhu and Bu 2006); **Hunan**: Dong’an, Hengyang, Sangzhi, Yanling, Yizhang, Yongshun (Ren and Xiong 1993), Zhangjiajie; **Jiangxi**: Jinggangshan, Jiulianshan, Yifeng, **Zhejiang**: Baishanzu (Bu, Zheng and Ren 1995), Lin’an, Longwangshan (Liu 1998), Tianmushan, Wuyanling (Fig. 16).

### ﻿ Manocoreus yunnanensis

Taxon classificationAnimaliaHemipteraCoreidae

Hsiao, 1964

2FEA2D03-E51B-58BC-9B2C-4DDBD5AC8E46

Figs 8D–F , 13D , 14H , 15H , 16

Manocoreus yunnanensis Hsiao, 1964: 91. Holotype: ♂, China, Yunnan, Xishuangbanna; IZCAS. Hsiao (1977): 244 (description, distribution, in key, photo); Ren (1983): 321 (in key), 323, 324 (figures); Zhu and Bu (2006): 239 (catalogue, distribution, description).

#### Type material examined.

***Holotype*** male labelled: “Yunnan: Xishuangbanna Mengna [printed in Chinese] / 550 [printed] Gongchi [printed in Chinese] / Chinese Academy of Sciences [printed in Chinese] // 1959•VI•30 [handwritten] / collector Zhang Facai [printed in Chinese] // IOZ(E) 221832 [printed] // HOLOTYPE [printed] // Manocoreus [handwritten] / yunnanensis [handwritten] / HSIAO [handwritten] / holotype Hsiao Tsaiyu identified [printed in Chinese] 19 [printed] 63 [handwritten]”; IZCAS. ***Paratype*** female, labelled: “NO. 59H [handwritten] Xishuangbanna [handwritten in Chinese] / Mengban [handwritten in Chinese] / 1958-6-9 [handwritten] // PARATYPE [printed] / Manocoreus [handwritten] / yunnanensis [handwritten] / HSIAO [handwritten]”; NKUM.

#### Other material examined.

**China. Yunnan**: Xishuangbanna Damenglong, 650 m a.s.l., 14.iv.1958, leg. Y.R. Zhang (1♂ IZCAS), same but 17.iv.1958, leg. F.J. Pu (1♂ IZCAS), Yunjinghong [= Jinghong], 650 m a.s.l., 8.viii.1958, leg. X.W. Meng (1♂ IZCAS), Jinghong, 30.ix.1979, leg. J.X. Cui (3♂♂ NKUM), Damenglong, 30.ix.1979, leg. H.G. Zou (1♀ NKUM).

#### Remarks.

This species is similar to *M.marginatus* in habitus, size, and color, but differs in the following characters: lateral margin of pronotum not black (Fig. 13D); punctures on the dorsum of head, pronotum, scutellum, and forewings not black (Fig. 8D); forewing concolorous; lateral side of the head, thorax and abdomen with blackish longitudinal stripe (Fig. 8E); distal portion of median ventroposterior process of the genital capsule with a small upward hook-shaped process in lateral view (see Ren 1983: fig. 26); plica of sternite VII not exposed out of sternite VI (Fig. 15H).

#### Distribution.

**China. Guizhou**: Fanjingshan (Zhu and Bu 2006); **Yunnan**: Xishuangbanna (Fig. 16).

### ﻿Key to species of *Manocoreus* Hsiao, 1964

**Table:** 

1	Connexivum without black spots (Fig. 7A)	**2**
–	Connexivum with black spots (Fig. 2C)	**3**
2	Subcostal margin of forewing black (Fig. 7A) (body length 13.5–15.5 mm)	***M.marginatus***
–	Subcostal margin of forewing not black (Figs 8A) (body length 12.1–14.2 mm)	***M.yunnanensis***
3	Bigger body size, > 15 mm (Fig. 1C–D) (body length 16.0–17.2 mm)	***M.furcatus***
–	Smaller body size, ≤ 16 mm	**4**
4	Middle of corium without black spot (Fig. 1A)	**5**
–	Middle of corium with black spot (Fig. 3A, C)	**6**
5	Labium surpassing anterior margin of metacoxae (Fig. 1B); median ventroposterior process of genital capsule triangular from ventral view (Fig. 14A) (body length 13.5–15.0 mm)	***M.astinus***
–	Labium not reaching anterior margin of metacoxae (Fig. 8B); median ventroposterior process of genital capsule long triangular from ventral view (Fig. 14G) (body length 12.5–14.0 mm)	***M.vulgaris***
6	Lateral margin of pronotum black (Figs 4A, 12D) (body length 12.02–14.95 mm)	***M.hsiaoi* sp. nov.**
–	Lateral margin of pronotum pale (Fig. 2A)	**7**
7	Antennomere III not dilated apically (body length 11.5–13.0 mm)	***M.grypidus***
–	Antennomere III dilated apically (body length 11.1–12.5 mm)	***M.montanus***

## ﻿Discussion

According to the only cladistic analysis based on morphological data which included Manocoreini, Manocoreini was the sister group of Gonocerini (Li 1997). Based on our examinations of specimens of these two tribes, Manocoreini has the following characteristics that are significantly different from the latter: (1) head with a small dentate or plate-like process in front of antenniferous tubercles, whereas without such process in Gonocerini; (2) plica of female sternum VII with triangular, or rectangular depression, covered by sternum VI, whereas the posterior margin of plica depressed and near the anterior margin of sternum VII in Gonocerini; (3) genital capsule has median ventroposterior process, whereas the posterior margin of genital capsule concave in Gonocerini; (4) basiconjunctivum moderately sclerotized, whereas strongly sclerotized in Gonocerini. According to recent seminal research on the morphology of spermatheca in Coreidae (Pluot-Sigwalt and Moulet 2020), the spermatheca of coreids was divided into three types (I, II, III), and type III could be subdivided into four subtypes (A, B, C, D). Based on our study, the spermatheca of Manocoreini belongs to type III, subtype A, and is closely related to Dasynini, Gonocerini, and Homoeocerini. Some recent molecular phylogenetic studies show that Manocoreini is closely related to Dasynini, Homoeocerini, Mictini, and Coreini (Ye et al. 2022) or Gonocerini and Coreini (Tian et al. 2023). Although Manocoreini can be clearly distinguished morphologically, their phylogenetic relationship still needs to be further verified by adding more molecular and biological evidences.

In the genus *Manocoreus*, *M.marginatus* and *M.yunnanensis* are closer in morphology than other species, such as having slender antenna (Figs 7A, B, 8D–F), posterior margin of male sternum VII concave ~ 1/3 in ventral view (Fig. 13E, H), and middle of female sternum VII sharply concave, both sides with process backward (Fig. 14E, H). More morphological and molecular evidences are needed to figure out the phylogenetic relationship and phylogeographical pattern of the species in this genus.

## Supplementary Material

XML Treatment for Manocoreini

XML Treatment for Manocoreus

XML Treatment for Manocoreus astinus

XML Treatment for Manocoreus furcatus

XML Treatment for Manocoreus grypidus

XML Treatment for Manocoreus hsiaoi

XML Treatment for Manocoreus marginatus

XML Treatment for Manocoreus montanus

XML Treatment for Manocoreus vulgaris

XML Treatment for Manocoreus yunnanensis
